# Education and Other Matters

**Published:** 1918-09-07

**Authors:** 


### A NURSES' CHARTER OF LIBERTY.

### Education and Other Matters.

It is commonly assumed, and possibly this may apply even to the College of Nursing, that the raising of the educational standard is a matter distinct from the economic problem. This is a fallacy. Not alone are the two interlaced, but the one is wholly dependent on the other. The offer of scholarships is a distinct symptom of the common desire after progress and enlightenment, but it would be a serious mistake to suppose that these alone, even if multiplied in number, can in any appreciable measure affect the education of the mass. Quite a different panacea is necessary to cure the existing ills of the profession. If reform is to ensue, the roots of the tree rather than the top branches must receive attention.

May I begin at the beginning—in other words, with the probationer? Can it be denied that the long hours enforced even in the best institutions, from the day that a young woman enters the Nursing Service, not only effectually kill all incentive to study, but tend to inspire a positive distaste for any such addition to the drudgery of life? I have a pathetic note from a sister in North London, who states that "it is impossible to study while on duty in the wards," a note that suggests much more than it expresses. After all, human endurance has its limits, and much toil, as well as much study, is a weariness to the flesh. Personally, I hold the view that "the waiting years"—the gap that separates the finished schoolgirl from the beginning of her career—is largely responsible for withering the zeal of the probationer to continue her pursuit after knowledge. For many reasons I should like to see an earlier age limit adopted, and a corresponding adaptation of the probationer's duties to suit her educational betterment rather than the financial requirements of the hospital. I should like to see the undergraduate nurse placed on a par, as regards years, with the woman medical undergraduate. But this is scarcely a practical proposition at present. It is, however, manifest that if the probationer's educational progress is to be seriously considered she must have opportunity and encouragement, and, above all, she should start having already a solid foundation laid. An eight-hour day would provide at least a part of the necessary opportunity. Suitable lectures, specified hours for study, and the necessary accommodation would provide the remainder. Then the College of Nursing, which is essentially, in one of its functions, an educational institution, might admit candidates of experience and attainments to Fellowships. Such a distinction would be as much coveted in the nursing world as in the medical.

It is, I believe, a thoroughly sound principle, but one which is often overlooked, that the only way to improve any profession is to begin at the lowest rung of the ladder. Improve the pay of the private soldier, and immediately or ultimately captain, major, and colonel will advance. Where the shoe really and seriously pinches is not in the first-class institution with its long waiting-list of candidates, but in those of second-rate status where the pay is bad and the work irregular and excessive. I wonder what educational progress the London Hospital authorities expect from probationers. "At present," says a correspondent from this institution, "the probationers and staff nurses work eleven hours on day duty. This does not include the three hours off duty, but it does include half an hour for midday dinner and half an hour for tea, the latter being taken in the wards." If this represents the model institution where the waiting-list is long, what is to be said for the hospitals of third-rate rank?

It would be idle for me to discuss the difficulties which public hospitals experience in finding ways and means. Suffice it to say that under the present *régime* these difficulties will never disappear or even diminish. The fundamental truth remains that the sick poor of the British nation are the *raison d'être* of the hospitals, be they of first-grade rank or third, and in making appeals for funds the claims of the nurse have never yet been put before the public. I can offer no opinion as to whether it is probable that if such claims were represented the public would suitably respond. In some cases they might, and in others otherwise. A Colonial nurse informs me that in some hospitals it is within her knowledge that an eight-hours day is in vogue, with one whole day off in seven, and she adds, with profound perception, that "people should be made to understand that this is the nurse's due," and that these conditions of employment should be made compulsory.

I am, I regret to observe, reluctantly driven to the conclusion that without State interference the emancipation of the nurse will never be achieved. The fact of supreme national importance is not the well-being of any profession, but the care of the sick poor, and there ought not to be in our midst in this twentieth century either second-class or third-class public hospitals. Government Inquiries and Blue-books demonstrate beyond question that there are both. I go further than these Inquiries and Reports, and I am prepared to assert and to prove that we have not yet arrived at a really first-class public hospital. Efficient nursing is wholly impossible where the junior drudge is obliged to work for eleven hours a day, and take her meals, or some of them, in the wards. I can quote instances from my own knowledge where nurses in so-called first-class hospitals have caused the death of their patients, and not through any fault of their own. Let me instance a case: A young man entered a hospital for treatment of a bone disease in his foot. He was attended by a nurse suffering from incipient influenza. He caught the infection, and within a week died from pneumonia. I can, if necessary, add a score of similar examples where worn-out women left in charge of the sick and suffering contributed to their decease. I have no doubt that every matron in the kingdom can do likewise.

To come back to education, I ask how is it possible to provide for the better education of overwrought toilers until their conditions of employment are completely changed? We must face the truth, although it may be unpalatable, and sooner or later the College of Nursing and every other champion of the profession will find that the economic problem surpasses every other in importance and urgency. Discipline, State Registration, and a One-Portal Examination are very useful in their place, but of much more far-reaching importance are the conditions of service. These should be made a first charge on every hospital, and if the desired end can be attained without State interference, so much the better. But can they? This is the problem which the executive of the College have to solve. It is a serious problem, but if fairly and squarely faced I believe that the nursing profession, the nursing press, and the British public will help towards its solution.

IERNE.

